# A meta-analysis of the association between diabetic patients and AVF failure in dialysis

**DOI:** 10.1080/0886022X.2018.1456464

**Published:** 2018-05-04

**Authors:** Yan Yan, Dan Ye, Liu Yang, Wen Ye, Dandan Zhan, Li Zhang, Jun Xiao, Yan Zeng, Qinkai Chen

**Affiliations:** a Department of Nephrology, The First Affiliated Hospital, Nanchang University, Nanchang, Jiangxi, People’s Republic of China;; b Jiangxi Medical College of Nanchang University, Jiangxi, People’s Republic of China

**Keywords:** Diabetes mellitus/diabetic, arteriovenous fistula/AVF, dialysis/hemodialysis, meta-analysis

## Abstract

**Purpose:** The most preferable vascular access for patients with end-stage renal failure needing hemodialysis is native arteriovenous fistula (AVF) on account of its access longevity, patient morbidity, hospitalization costs, lower risks of infection and fewer incidence of thrombotic complications. Meanwhile, according to National Kidney Foundation (NKF)/Dialysis Out-comes Quality Initiative (DOQI) guidelines, AVF is more used than before. However, a significant percentage of AVF fails to support dialysis therapy due to lack of adequate maturity. Among all factors, the presence of diabetes mellitus was shown to be one of the risk factors for the development of vascular access failure by some authors. Therefore, this review evaluates the current evidence concerning the correlation of diabetes and AVF failure.

**Methods:** A search was conducted using MEDLINE, SCIENCE DIRECT, SPRINGER, WILEY-BLACKWELL, KARGER, EMbase, CNKI and WanFang Data from the establishment time of databases to January 2016. The analysis involved studies that contained subgroups of diabetic patients and compared their outcomes with those of non-diabetic adults. In total, 23 articles were retrieved and included in the review.

**Results:** The meta-analysis revealed a statistically significantly higher rate of AVF failure in diabetic patients compared with non-diabetic patients (OR = 1.682; 95% CI, 1.429–1.981, Test of OR = 1: *z* = 6.25, *p* <.001).

**Conclusions:** This review found an increased risk of AVF failure in diabetes patients. If confirmed by further prospective studies, preventive measure should be considered when planning AVF in diabetic patients.

## Introduction

The number of patients with chronic kidney disease (CKD) as well as those with end-stage renal disease (ESRD) is on the rise worldwide. Under the new updated guidance of National Kidney Foundation (NKF)/Dialysis Out-comes Quality Initiative (DOQI) guidelines [1]. Arteriovenous fistula (AVF) is the most preferred choice for chronic hemodialysis (HD) among the three main types of access [i.e., AVF, arteriovenous graft, and central venous catheter (CVC)]. Meanwhile, there are a number of researchers reported the diabetes mellitus may be associated with arteriovenous fistula failure, and the effects of diabetes on overall AVF outcomes may be minimized by careful preoperative vessel imaging and AVF site selection. Therefore, this review was aimed to find out whether diabetes was related with AVF failure by meta-analysis.

## Methods

This systematic review and meta-analysis were conducted according to the Preferred Reporting Items for Systematic Review and Meta-Analysis (PRISMA) guidelines [2].

**Figure 1.**
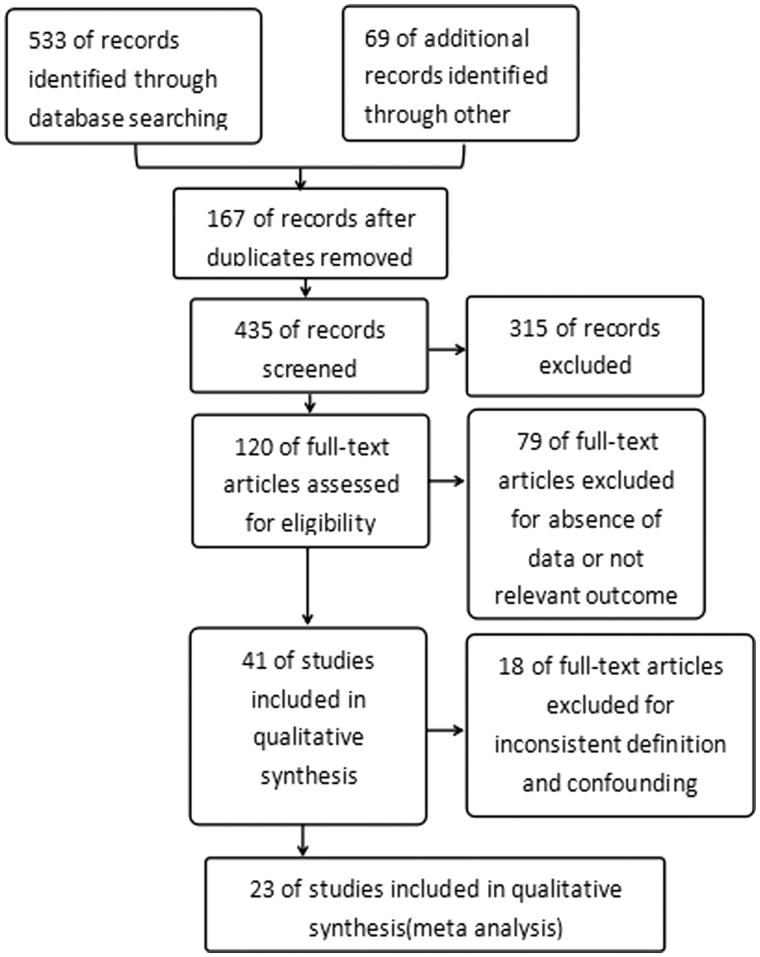
Study flow diagram of the meta-analysis.

**Search strategy.** We performed an online search published in English and Chinese using the electronic databases MEDLINE, SCIENCE DIRECT, SPRINGER, WILEY-BLACKWELL, KARGER, EMbase, CNKI and WanFang Data by the University library resource discovery system for relevant retrospective studies and prospective studies up to January 2016. The keyword combination is ‘((arteriovenous fistula failure) OR arteriovenous fistula thrombosis) AND diabetes mellitus’. After obtaining the results, 553 (388 Chinese, 165 English respectively) abstracts of the relevant titles had been read online, 69 relevant articles (53 English, 16 Chinese) were checked by full text. The references of the identified articles were resolved as well. Eventually, we selected 120 articles and read through (Figure 1).

**Selection criteria.** (1) We included studies concerning risk factors for arteriovenous fistula failure in dialysis patients up to January 2016; (2) research methods: retrospective and prospective studies; (3) the definition of exposure factor is similar and no difference of diagnostic criteria of AVF failure and diabetes; (4) there was no restriction with regard to publication status; language was confined to English and Chinese; (5) there were control group and diabetic group.

**Exclusion criteria.** (1) Repeated reports, poor quality, little information or absent data; (2) inconsistent definitions and included confounding factors. (3) Different diagnosis criteria, animal experiments and qualitative research. (4) Review articles and case reports would be excluded from the analysis.

**About definitions.** Diabetes mellitus was considered to be present if the patient was on a diabetic diet, current use of hypoglycemic medication, use of insulin or when the diagnosis was recorded in a medical status, or if the fasting plasma glucose exceeded 140 mg/dl. AVF failure was defined as primary non-function due to thrombosis (before puncturing) or non-maturation [3]. Vascular access thrombosis (VAT) was defined as a lack of blood flow diagnosed by palpation and auscultation. The presence of thrombus was confirmed by surgery or angiography.

Twenty-three relevant studies were identified using the above criteria and included in the final analysis (Table 1) [3–25]. The types of research used in the meta-analysis included retrospective and prospective studies; as expected, there was not any randomized controlled trial. The prespecified study end points were thrombosis or failure to mature resulting in inadequate functioning.

**Table 1.** Twenty-three Studies included in qualitative synthesis.

study	year	race	tevent	tnoevent	ttotal	cevent	cnoevent	ctotal	sum
Shen B	2006	Asian	4	11	15	2	23	25	40
Zhang ZM	2007	Asian	18	58	76	57	303	360	436
Liao YL	2015	Asian	10	13	23	21	74	95	118
Jiang Y	2013	Asian	7	25	32	11	29	40	72
Hu DJ	2014	Asian	14	20	34	6	28	34	68
Zhang YL	2010	Asian	12	20	32	56	154	210	242
Baris Afsar Rengin Elsurer	2012	Caucasian	30	44	74	43	116	159	233
Najiba Fekih-Mrissa	2011	Caucasian	9	19	28	17	33	50	78
Jamshid Roozbeh	2005	Caucasian	16	15	31	68	72	140	171
Ju-Young Moon	2015	Asian	70	39	109	230	139	369	478
BRANED J.MANNS	1998	Caucasian	9	18	27	23	39	62	89
DOUGLAS SHEMIN	1999	Caucasian	28	19	47	15	22	37	84
A.Bahadi	2012	Caucasian	14	19	33	32	73	105	138
Engin Usta	2012	Caucasian	11	7	18	41	21	62	80
Yao SL 2015	2015	Asian	13	18	31	43	141	184	215
Li ZZ 2014	2014	Asian	28	45	73	30	106	136	209
Wang YZ	2005	Asian	9	13	22	6	30	36	58
Zhong HY	2010	Asian	10	13	23	22	83	105	128
Chen MX11	2015	Asian	8	18	26	24	137	161	187
Wei XH	2015	Asian	6	15	21	22	53	75	96
Cui TL	2012	Asian	33	30	63	68	135	203	266
He Q	2009	Asian	9	25	34	35	280	315	349
Ramazan Danis	2009	Caucasian	17	41	58	29	145	174	232

**Statistical analysis.** Data were extracted from the studies and recorded into Table 1. AVF failure was transformed into a dichotomous outcome. Data extraction was done from tables, texts, or graphs from relevant sources. Statistical analysis was performed using STATA 12.0 software and Review Manager 5.3 software.

**Forest plot**: The odds ratio (OR) was used to present effects in a logarithmic scale in the analysis. An OR >1 indicated higher risk in diabetes patients, whereas an OR <1 indicated higher risk in non-diabetes patients. The 95% confidence interval (CI) describes the possible range that the pooled OR could take; any CI that included 1 (the point of equal effect of the two groups) was considered not to be statistically significant. In the analysis, the line of identity is 1 and the CI is significant if it does not cross 1. The OR and 95% CI for combined studies were calculated using the M-H heterogeneity model of meta-analysis. The presence of heterogeneity was assessed using the I^2^ statistic.

**Funnel plot:** (1) Use effects estimated value as the abscissa and sample size as the ordinate to draw a scatter plot. According to the degree of asymmetric graphics to judge whether there are bias or not in the meta analysis; (2) based on the fact that the accuracy of the effects is proportional to the sample size. In the analysis, a funnel plot for all studies was included to assess the degree of publication bias, any asymmetry around the vertical axis indicated the presence of such bias.

## Results

**Figure 2.**
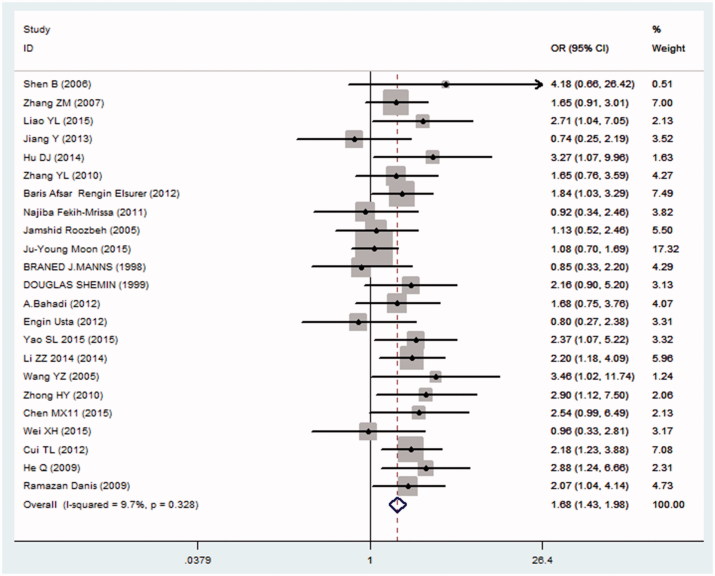
Forest plot.

Twenty-three studies were used in the analysis, which included a total of 930 diabetic patients and 3137 nondiabetic patients with end-stage renal disease. AVF failure was reported in all studies (Table 1). The pooled OR estimate for the AVF failure was 1.682 (95% CI, 1.429–1.981, Test of OR = 1: z = 6.25, *p* < .001) in favor of the diabetic patients (Figure 2). These results revealed a statistically significantly higher rate of AVF failure in diabetic patients compared with nondiabetic patients.

**Figure 3.**
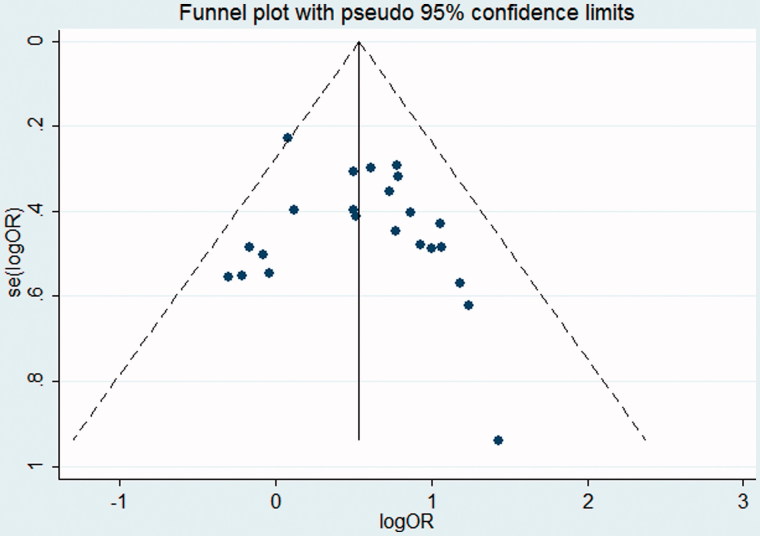
Funnel plot with pseudo 95% confidence limits.

**Figure 4.**
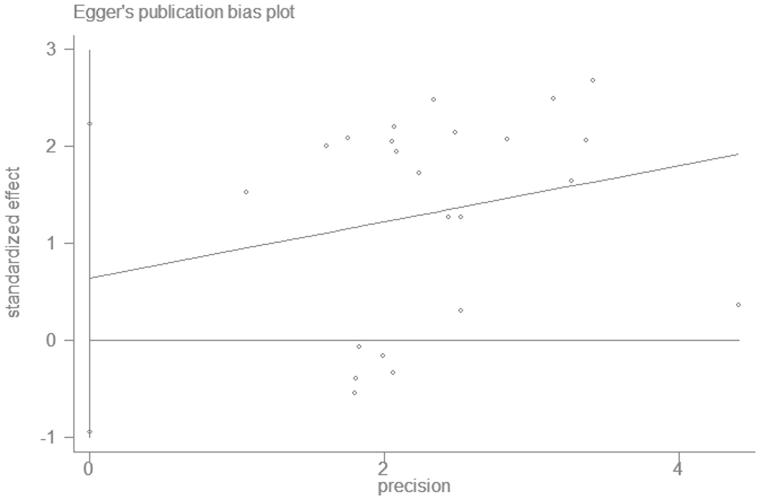
Begg’s funnel plot.

**Figure 5.**
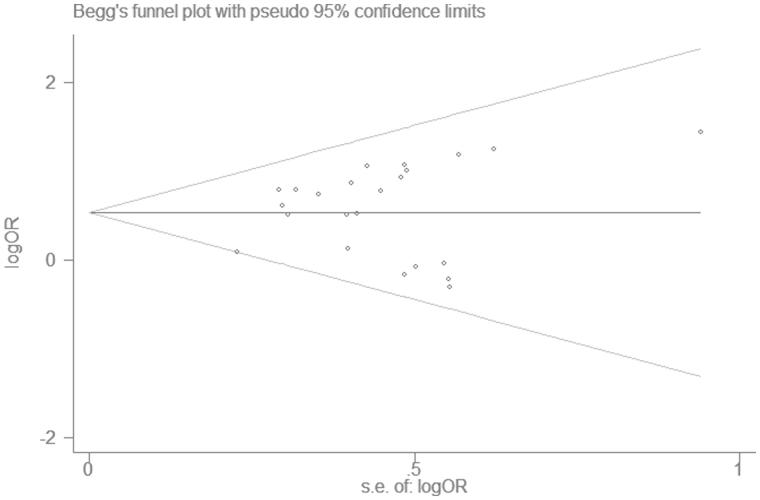
Egger’s funnel plot.

A funnel plot shows publication bias of the studies, as seen from the Figure 3, the plot was over-all symmetrical, which indicates little publication bias. The Begg’s test and Egger’s test were also used to check publication bias quantitatively. Begg’s test *p* = .526 > .05 (Figure 4) and Egger’s test *p* = .408 > .05 (Figure 5), implies little publication bias. As shown in the Figure 2, there was almost non-heterogeneous between the studies (I^2^=9.7%; Figure 2).

## Discussion

AVF is the main permanent vascular access for hemodialysis and a lifeline for patients who need hemodialysis. Mature AVF provides adequate blood flow and long working time for hemodialysis with few complications and has become the ideal vascular access for patients receiving hemodialysis. But, AVF dysfunction is still the primary problem bothering doctors and patients.

As the changes of the primary disease, the number of diabetic patients is on the rise. Besides, the presence of diabetes is apt to diabetic nephropathy, which commonly needs a hemodialysis therapy at the end [26]. The results from the meta-analysis study indicate that diabetes patients are more likely to cause AVF failure. Although the mechanism is not clear yet, the following may provide a possible explanation. Diabetes causes a high risk of platelet aggregation and increases release of von Willebrand factor, which promotes the platelet aggregation and results in damaging to endothelial cells in blood vessels [27]. The pathophysiological mechanism of diabetes leads to the formation of thrombosis more easily. If vascular intima damage and angiosclerosis exist simultaneously, thrombosis formation becomes extremely easily. Basic research proves that the blood vessel walls have both the antithrombotic and prothrombotic factors the ultimate consequences after vascular injury depends on the balance of the two sides. Besides, hyperglycemia and the increasing of glycosylation end products bring about a series of bioactive substances disorders, making it liable to injure to the internal wall of vessels and blood vessels become less elastic, thereby leading to blood flow retardation and platelet aggregation, and ultimately thrombosis.

On account of atherosclerosis exists more often and severe in diabetes patients, there is a wide range of vascular lesions, making it difficult to establish a vascular access [28]. In addition, diabetes is often accompanied by hyperlipemia, hypoalbuminemia, and high blood coagulation, which also lead to AVF’s obstruction. beyond that, diabetic patients prone to lipid deposition, especially in vascular wall near anastomotic stoma, causing dysplasia of blood clots or postoperative fistula more easily. Due to hemodynamics effects, the vein which near anastomotic stoma is struck by blood flow, leading to inner membrane injury, platelets and fiberdeposition, causing vascular intimal hyperplasia and stenosis.

Some studies indicated atherosclerotic changes in forearm arteries in diabetic patients appear in 60% of hemodialysis patients [28]. It is possible to create a native AVF in 90% of diabetic patients, although this requires more procedures. However, it is difficult to create an AVF only in the remaining 10% of diabetic patients. In patients with diabetes, AVF in the wrist region should be preferred [28]. In case of fistula failure, revision is a reliable procedure salvaging a failed fistula, which yields an acceptable patency rate of regardless of the patient’s risk factors for arteriosclerosis [29].

In order to make AVF work longer, following precautionary measures should be taken: (1) blood glucose control; (2) weight control, adjust blood lipid metabolism; (3) strengthen the fistula in exercise after establishing fistulas [30]; (4) timing monitoring vascular access, improve the rate of nutritional status, diabetes control; (5) recommend peritoneal dialysis in diabetes patients.
